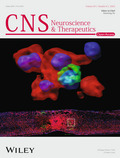# Front cover

**DOI:** 10.1111/cns.14375

**Published:** 2023-07-18

## Abstract

The cover image is based on the Original Article *Regulatory T cells promote functional recovery after spinal cord injury by alleviating microglia inflammation via STAT3 inhibition* by Rui Liu et al., https://doi.org/10.1111/cns.14161.